# Insulin resistance assessed by estimated glucose disposal rate and the risk of abdominal aortic calcification: findings from a nationwide cohort study

**DOI:** 10.3389/fendo.2025.1560577

**Published:** 2025-05-12

**Authors:** Bo Zhao, Zongliang Yu, Fengyan Tang, Zhenqin Feng, Junfeng Wang, Zhaoxiang Wang

**Affiliations:** ^1^ Department of Cardiology, Affiliated Kunshan Hospital of Jiangsu University, Kunshan, Jiangsu, China; ^2^ Department of Endocrinology, Affiliated Kunshan Hospital of Jiangsu University, Kunshan, Jiangsu, China

**Keywords:** ABDOMINAL AORTIC CALCIFICATION, insulin resistance, estimated glucose disposal rate, NHANES, cross-sectional study

## Abstract

**Purpose:**

The estimated glucose disposal rate (eGDR) serves as a straightforward and noninvasive indicator of insulin resistance (IR). This study aims to explore the association between eGDR and the risk of abdominal aortic calcification (AAC).

**Methods:**

We utilized data from adult participants (≥40 years old, n=3006) from the 2013–2014 National Health and Nutrition Examination Survey (NHANES) database. AAC was measured by dual-energy X-ray absorptiometry and quantified using the Kauppila score. Severe AAC (SAAC) was defined as an AAC score > 6. Logistic regression, restricted cubic spline (RCS), and subgroup analysis were used to analyze the relationship between eGDR and SAAC risk.

**Results:**

In fully adjusted models, eGDR was found to be negatively associated with SAAC (OR=0.86, 95%CI:0.79–0.94, *P*<0.001). Compared to participants in the lowest eGDR quantile, those in the highest quantile exhibited a lower risk of SAAC (OR=0.47, 95%CI:0.25–0.91, *P*=0.026). The RCS analysis indicates a nonlinear relationship between eGDR and SAAC risk, with a turning point at 7.05 mg/kg/min. Subgroup analysis showed that the association between eGDR and SAAC risk was more significant in women.

**Conclusions:**

The degree of IR assessed by eGDR is associated with SAAC risk. The eGDR shows promise as an epidemiological tool for evaluating the influence of IR on AAC.

## 1 Introduction

Abdominal aortic calcification (AAC) is a common vascular lesion characterized by the deposition of calcium salts on the abdominal aorta walls, leading to stiffening and reduced elasticity of the aortic walls (1). AAC is commonly detected through imaging techniques such as X-ray and computed tomography (CT) (2, 3). Numerous studies have demonstrated that its degree of calcification is closely associated with the risk of cardiovascular diseases (4). AAC is prevalent in the general population and is closely associated with a variety of factors (5). Metabolic conditions such as hyperlipidemia, diabetes, hypertension, and metabolic syndrome significantly contribute to its progression (4, 6–8).

As an early warning signal of disease, identifying the risk factors for AAC is especially important for preventing its progression. Insulin resistance (IR), a key feature of metabolic dysfunction, refers to a clinical condition characterized by reduced sensitivity and responsiveness to insulin (9). The presence of IR accelerates the progression of AAC, the severity of AAC could also reflect the cumulative effects of metabolic disturbances associated with IR (10, 11). While the hyperinsulinemic-euglycemic clamp remains the gold standard for detecting IR, its use in large-scale epidemiological studies is limited due to its demanding and time-intensive process (12). Recently, estimated glucose disposal rate (eGDR) has emerged as a simpler and reliable surrogate marker for IR (13). Calculated using hemoglobin A1c (HbA1c), waist circumference (WC), and hypertension status, the eGDR demonstrates an inverse association with the severity of IR (14). Prior research has also demonstrated a link between eGDR levels and the risk of cardiovascular events and mortality (15–18).

However, it remains unclear whether eGDR can effectively identify individuals at higher risk of AAC. Therefore, we aim to investigate this association in the U.S. general population using data from the National Health and Nutrition Examination Survey (NHANES) database. We hypothesize that higher eGDR levels are negatively associated with the severity of AAC.

Recent studies on IR and vascular calcification have predominantly relied on traditional markers such as the Homeostasis Model Assessment of Insulin Resistance (HOMA-IR) and the Triglyceride-Glucose (TyG) index (10, 19, 20). However, these metrics may not adequately capture the full metabolic complexity of IR. Evidence from prior research has demonstrated that the eGDR exhibits superior predictive capabilities when compared to HOMA-IR and the TyG index (21–23). Therefore, this study leverages eGDR, a comprehensive metric that integrates key components of metabolic syndrome, including glycemic control, central obesity, and hypertension. Utilizing data from the nationally representative National Health and Nutrition Examination Survey (NHANES) cohort, this investigation seeks to establish eGDR as a valuable tool for AAC risk stratification, thereby informing more effective and targeted prevention strategies.

## 2 Materials and methods

### 2.1 Study population

Data for this study were drawn from NHANES, a survey conducted by the National Center for Health Statistics at the Centers for Disease Control and Prevention. The survey used a stratified, randomized, multi-stage sampling approach to ensure a nationally representative sample. Participants underwent physical examinations, completed health and nutrition surveys, and participated in laboratory tests. The NHANES protocol was reviewed and approved by the Ethics Review Board of the National Center for Health Statistics (NCHS), and written informed consent was collected from all participants (https://www.cdc.gov/nchs/nhanes/irba98.htm). Detailed methodologies and datasets are available at https://www.cdc.gov/nchs/nhanes/. This study focused on NHANES 2013–2014 data, selecting 3006 participants aged 40 or older with complete eGDR and AAC data.

### 2.2 Definition of eGDR and SAAC

The eGDR (mg/kg/min) serves as an indicator of IR and is determined using the following formula: eGDR = 21.158 − (0.09 × WC) − (3.407 × HTN) − (0.551 × HbA1c), where WC represents waist circumference (cm), HTN indicates hypertension status (yes = 1, no = 0), and HbA1c refers to glycated hemoglobin (%) (16). The AAC severity was assessed using the AAC score system, which was exclusively derived from the NHANES 2013–2014 dataset. This score, based on the Kauppila scoring method and measured through dual-energy X-ray absorptiometry (DXA, Densitometer Discovery A, Hologic, Marlborough, MA, USA), ranged from 0 to 24, with higher values indicating more severe calcification. An AAC score exceeding 6 was classified as severe AAC (SAAC) (24). Both the AAC scores, and SAAC were utilized as outcome variables in this research.

### 2.3 Assessment of covariates

The covariates considered in this study included demographic factors (age, gender, race), socio-economic variables (marital status, income, education), smoking history, health conditions (hypertension, diabetes, cardiovascular disease), body mass index (BMI), WC, HbA1c, bone metabolism (serum calcium, phosphorus, total 25-hydroxyvitamin D), serum creatinine (Scr), and estimated glomerular filtration rate (eGFR). The eGFR was estimated using the Chronic Kidney Disease Epidemiology Collaboration (CKD-EPI) equation, which incorporates age, gender, race, and Scr levels (25). Diabetes was determined through self-reported physician diagnoses, fasting plasma glucose (FPG) levels of ≥7.0 mmol/L, HbA1c levels of ≥6.5%, or the use of antidiabetic drugs. Hypertension was defined based on self-reported physician diagnosis, systolic blood pressure (SBP) ≥140 mmHg, diastolic blood pressure (DBP) ≥90 mmHg, or the use of antihypertensive medications. Cardiovascular diseases were identified based on participants’ self-reported histories of heart attacks, strokes, heart failure, coronary artery disease, or angina.

### 2.4 Statistical analysis

Statistical analyses adhered to the Centers for Disease Control and Prevention guidelines (CDC) (https://wwwn.cdc.gov/nchs/nhanes/analyticguidelines.aspx), utilizing a complex multistage cluster survey design, and incorporating weights. Continuous variables were presented as mean and 95% confidence interval (CI), while categorical variables were expressed as percentages and 95% CI. Group differences in continuous and categorical variables were evaluated using the weighted Student’s t-test and chi-squared test, respectively. Logistic and linear regression models were employed to examine the association between eGDR and SAAC and AAC scores. Nonlinear relationship between eGDR and SAAC was explored using restricted cubic spline (RCS) analysis. A two-piecewise regression model was employed to identify intervals, and the Log-likelihood ratio test was utilized to assess the existence of a threshold. Subgroup analyses were conducted based on age, gender, race, marital status, smoking, annual household income, education level, BMI, diabetes, and cardiovascular disease, with adjustments for other covariates. Receiver Operating Characteristic (ROC) analysis was conducted to evaluate and compare the performance of eGDR and other insulin resistance (IR) indices, including Metabolic Score for Insulin Resistance (METS-IR), TyG, HOMA-IR, and Triglyceride-to-High-Density Lipoprotein Cholesterol Ratio (TG/HDL-c) in assessing SAAC risk. All statistical analyses in this research were performed using Empower software (http://www.empowerstats.com) and R software (http://www.R-project.org), with a two-sided *P* value < 0.05 considered statistically significant.

## 3 Results

### 3.1 Baseline characteristics of study population

The study included 3006 participants, with a mean age of 57.43 years. The racial composition was 6.92% Mexican Americans, 9.93% Non-Hispanic Blacks, 71.32% Non-Hispanic Whites, 4.68% Other Hispanics, and 7.15% from other racial groups. Of these, 270 participants were diagnosed with SAAC. A weighted analysis was performed to compare the general and clinical characteristics of participants with and without SAAC (**Table 1**). Results demonstrated that participants with SAAC tended to be older, unmarried, smokers, and had higher rates of diabetes, hypertension, cardiovascular disease, HbA1c, Scr, total 25-hydroxyvitamin D, and AAC scores (*P*<0.01). In contrast, they showed reduced levels of BMI, eGFR, educational attainment, and annual household income (*P*<0.01). The differences in racial distribution were also statistically significant (*P*<0.001). Notably, eGDR levels were reduced in the SAAC group compared to the non-SAAC group (*P*<0.001).

**Table 1:** Baseline characteristics of the study population.

Characteristics	Overall (n=3006)	Non-SAAC (n=2736)	SAAC (n=270)	*P* value
Age (years)	57.43 (56.86, 58.00)	56.27 (55.79, 56.75)	71.07 (69.89, 72.26)	<0.001
Gender				0.462
Female	51.68 (49.92, 53.45)	51.40 (49.28, 53.51)	55.08 (45.44, 64.35)	
Male	48.32 (46.55, 50.08)	48.60 (46.49, 50.72)	44.92 (35.65, 54.56)	
Race, %				0.003
Mexican American	6.92 (4.17, 11.26)	7.16 (4.32, 11.62)	4.10 (1.83, 8.91)	
Non-Hispanic Black	9.93 (7.42, 13.16)	10.25 (7.59, 13.69)	6.18 (4.17, 9.06)	
Non-Hispanic White	71.32 (64.35, 77.42)	70.58 (63.24, 76.98)	80.15 (73.10, 85.72)	
Other Hispanic	4.68 (3.15, 6.90)	4.87 (3.29, 7.16)	2.44 (1.03, 5.66)	
Other Races	7.15 (5.70, 8.93)	7.15 (5.71, 8.91)	7.14 (3.95, 12.56)	
Married, %	65.97 (62.96, 68.85)	67.57 (64.53, 70.46)	47.11 (40.39, 53.93)	<0.001
Annual household income (under $20000), %	13.08 (9.19, 18.30)	12.29 (8.45, 17.55)	22.32 (16.09, 30.09)	<0.001
Education level (above high school), %	63.01 (57.26, 68.40)	63.81 (58.24, 69.03)	53.55 (43.18, 63.62)	0.006
Smoking history, %	45.79 (42.22, 49.41)	44.39 (40.93, 47.90)	62.32 (53.40, 70.47)	<0.001
Diabetes, %	16.86 (15.09, 18.80)	15.29 (13.52, 17.26)	35.38 (28.36, 43.10)	<0.001
Hypertension, %	49.28 (47.18, 51.39)	46.90 (44.73, 49.07)	77.42 (71.63, 82.32)	<0.001
Cardiovascular disease, %	11.08 (9.62, 12.73)	9.57 (8.23, 11.09)	28.92 (22.47, 36.35)	<0.001
BMI (kg/m^2^)	28.51 (28.16, 28.86)	28.62 (28.28, 28.96)	27.21 (26.27, 28.14)	0.006
WC (cm)	99.79 (99.11, 100.48)	99.86 (99.19, 100.53)	99.01 (97.14, 100.89)	0.362
HbA1c (%)	5.77 (5.72, 5.83)	5.74 (5.68, 5.80)	6.17 (6.01, 6.33)	<0.001
Scr (µmol/L)	81.82 (80.44, 83.20)	80.99 (79.37, 82.62)	91.53 (86.57, 96.48)	0.003
eGFR (mL/min/1.73m²)	84.64 (83.75, 85.54)	85.95 (84.96, 86.94)	69.25 (66.74, 71.76)	<0.001
Serum calcium (mg/dL)	9.46 (9.43, 9.48)	9.45 (9.43, 9.48)	9.48 (9.43, 9.54)	0.248
Serum phosphorus (mg/dL)	3.80 (3.76, 3.83)	3.79 (3.76, 3.82)	3.87 (3.78, 3.96)	0.086
Total 25-hydroxyvitamin D (nmol/L)	74.94 (72.25, 77.63)	74.31 (71.42, 77.20)	82.41 (78.80, 86.02)	<0.001
AAC score	1.47 (1.27, 1.67)	0.66 (0.57, 0.74)	11.04 (10.51, 11.57)	<0.001
eGDR (mg/kg/min)	7.32 (7.20, 7.43)	7.41 (7.29, 7.53)	6.21 (5.96, 6.46)	<0.001

BMI, body mass index; WC, waist circumference; HbA1c, hemoglobin A1c; Scr, serum creatinine; eGFR, estimated glomerular filtration rate; AAC, abdominal aortic calcification; eGDR, estimated glucose disposal rate.

### 3.2 The relationship between eGDR and AAC risk

A significant negative association between eGDR and SAAC risk was identified through logistic regression analysis (**Table 2**) (*P*<0.05). Without adjusting for covariates, higher eGDR levels were linked to a decreased likelihood of SAAC (OR=0.84, 95%CI:0.80–0.89, *P*<0.001). This association persisted after covariate adjustments in Model 2 and Model 3, with ORs of 0.94 and 0.86, respectively (*P*<0.05). After fully adjusting for confounders and stratifying eGDR into quartiles (Q1 to Q4), the risk of SAAC showed a progressive increase of 48% in Q2, while it decreased by 15% and 53% in Q3 and Q4, respectively (*P* for trend<0.01). Linear regression analysis also showed a correlation between eGDR levels and AAC scores (β=-0.14, 95%CI: -0.21–0.07, *P*<0.001) (**Table 3**).

**Table 2:** Logistic regression analysis results of eGDR and SAAC.

SAAC, OR (95%CI), *P* value			
Variables	Model 1	Model 2	Model 3
eGDR	0.84 (0.80, 0.89) <0.001	0.94 (0.88, 1.00) 0.042	0.86 (0.79, 0.94) <0.001
Quartiles			
Q1	reference	reference	reference
Q2	1.50 (1.11, 2.04) 0.009	1.65 (1.16, 2.34) 0.005	1.48 (0.97, 2.27) 0.072
Q3	0.68 (0.47, 0.97) 0.032	1.02 (0.68, 1.52) 0.921	0.85 (0.52, 1.37) 0.495
Q4	0.23 (0.14, 0.38) <0.001	0.60 (0.35, 1.04) 0.068	0.47 (0.25, 0.91) 0.026
*P* for trend	<0.001	0.093	0.004

OR, odds ratio.

95% CI: 95% confidence interval.

**Model 1:** no covariates were adjusted.

**Model 2:** adjusted for adjusted for age, gender, race, marital status, income, education, and smoking.

**Model 3:** further adjusted for adjusted for diabetes, cardiovascular diseases, BMI, Scr, eGFR, serum calcium, serum phosphorus, and total 25-hydroxyvitamin D based on the model 2.

**Table 3:** Linear regression analysis results of eGDR and AAC scores.

AAC scores, β (95%CI), *P* value			
Variables	Model 1	Model 2	Model 3
eGDR	-0.21 (-0.26, -0.16) <0.001	-0.05 (-0.10, -0.00) 0.041	-0.14 (-0.21, -0.07) <0.001
Quartiles			
Q1	reference	reference	reference
Q2	0.52 (0.18, 0.87) 0.003	0.59 (0.25, 0.92) <0.001	0.37 (-0.01, 0.74) 0.060
Q3	-0.52 (-0.87, -0.17) 0.004	0.03 (-0.31, 0.36) 0.878	-0.17 (-0.55, 0.20) 0.369
Q4	-1.27 (-1.62, -0.92) <0.001	-0.25 (-0.60, 0.10) 0.163	-0.71 (-1.17, -0.24) 0.003
*P* for trend	<0.001	0.032	<0.001

95% CI: 95% confidence interval.

**Model 1:** no covariates were adjusted.

**Model 2:** adjusted for adjusted for age, gender, race, marital status, income, education, and smoking.

**Model 3:** further adjusted for adjusted for diabetes, cardiovascular diseases, BMI, Scr, eGFR, serum calcium, serum phosphorus, and total 25-hydroxyvitamin D based on the model 2.

### 3.3 RCS analysis

**Figure 1:**
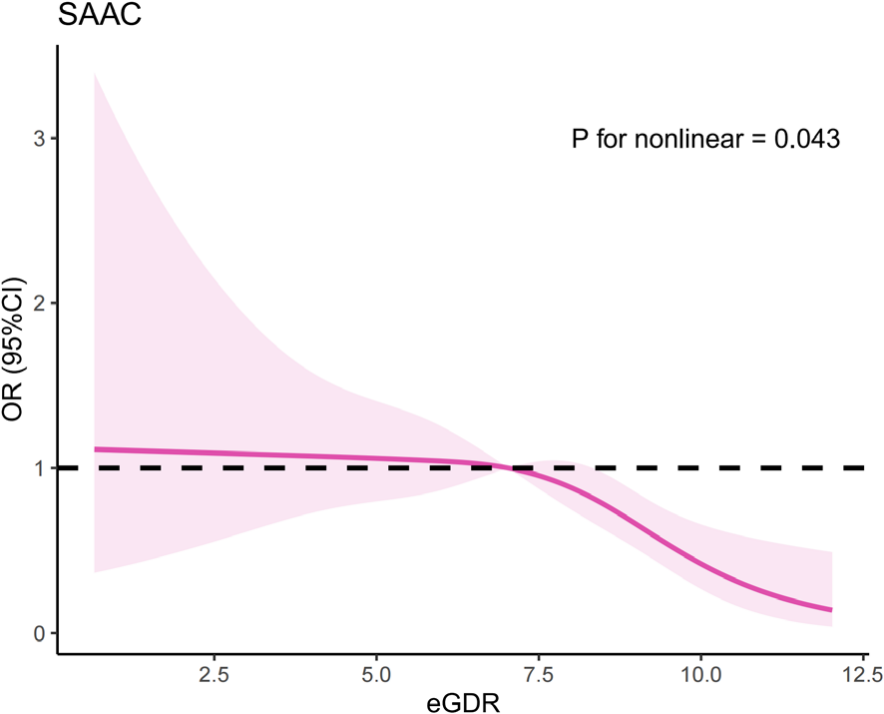
The results of RCS analysis.

Multivariate logistic regression analysis indicated there is a potential nonlinear relationship between eGDR and SAAC risk. To further explore this association, we conducted an RCS analysis, which revealed an inverted U-shaped relationship (**Figure 1**) (P for nonlinear=0.043). We also employed segmented regression analyses to investigate the association between eGDR and SAAC risk, identifying a threshold value of 7.05 mg/kg/min for eGDR (**Table 4**). When the eGDR exceeded 7.05 mg/kg/min, each 1-unit increase in eGDR was associated with a 28% reduction in the risk of SAAC (OR=0.72, 95%CI:0.61–0.85, P<0.001). When the eGDR was below 7.05 mg/kg/min, no notable correlation with SAAC risk could be identified (P=0.771).

**Table 4:** Threshold effect analysis of eGDR on SAAC risk.

Model	OR (95% CI) *P* value
Total	0.86 (0.79, 0.94) <0.001
Breakpoint (K)	7.05
OR1 (<7.05)	1.02 (0.88, 1.20) 0.771
OR2 (>7.05)	0.72 (0.61, 0.85) <0.001
OR2/OR1	0.70 (0.54, 0.91) 0.009
*P* for logarithmic likelihood ratio	<0.001

adjusted for adjusted for age, gender, race, marital status, income, education, and smoking, diabetes, cardiovascular diseases, BMI, Scr, eGFR, serum calcium, serum phosphorus, and total 25-hydroxyvitamin D.

### 3.4 Subgroup analyses

**Figure 2:**
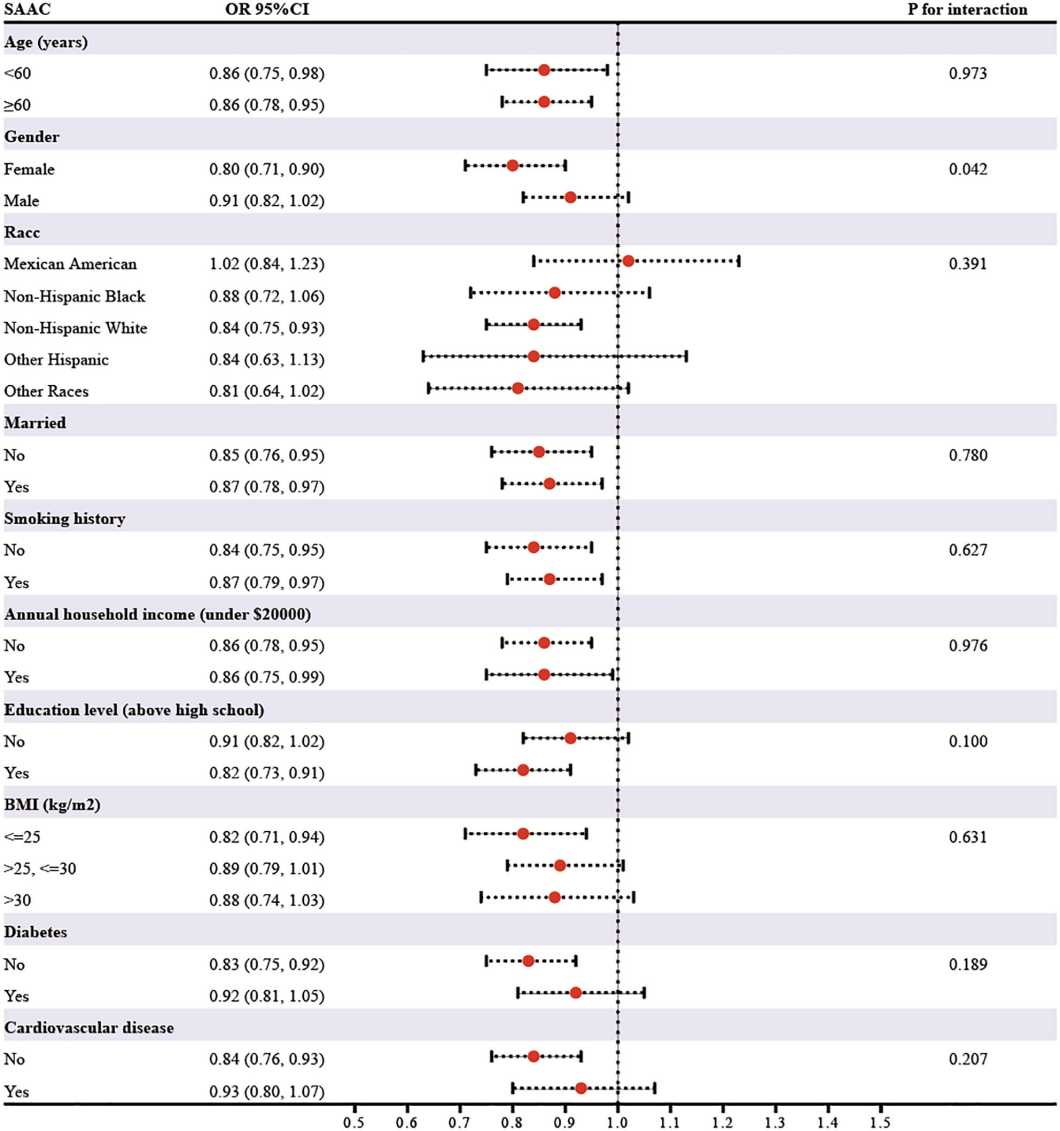
The results of subgroup analysis.

In subgroup analyses stratified by age, gender, race, marital status, smoking, annual household income, education level, BMI, diabetes, and cardiovascular disease, the relationship between eGDR on SAAC risk appeared to be more pronounced in females than in males (*P* for interaction=0.042) (**Figure 2**). Additionally, the relationship remained consistent across other subgroups defined by the other variables (*P* for interaction>0.05).

### 3.5 ROC analysis

**Figure 3:**
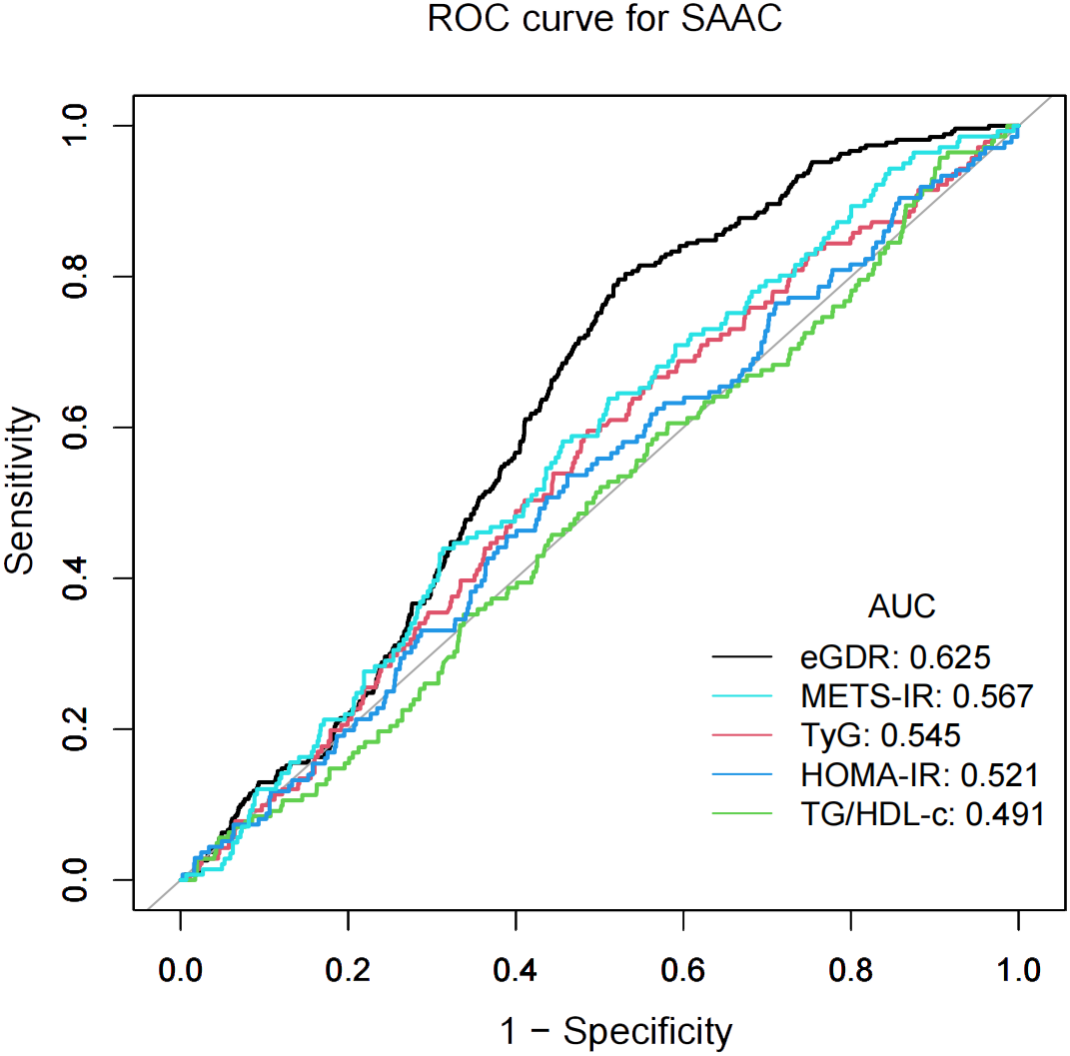
The results of ROC analysis.

The predictive accuracy of eGDR and other IR indices (METS-IR, TyG, HOMA-IR, and TG/HDL-c) for SAAC risk was evaluated using ROC curve analyses, as shown in **Figure 3**. METS-IR was calculated as Ln [(2× fasting plasma glucose × triglycerides × BMI)/high density lipoprotein-cholesterol] (26), and the TyG index as Ln [triglycerides × fasting plasma glucose/2] (20). HOMA-IR was computed as fasting plasma glucose × fasting insulin/22.5 (27). The TG/HDL-c was calculated by dividing triglycerides by high density lipoprotein-cholesterol (28). Compared to other IR indices, eGDR showed higher performance power for SAAC risk. The area under the curve (AUC) values were: eGDR, 62.5%; METS-IR, 56.7%; TyG, 54.5%; HOMA-IR, 52.1%; and TG/HDL-c, 49.1%.

## 4 Discussion

In this cross-sectional study, we found that higher eGDR levels were associated with a reduced risk of SAAC in the general population of the United States. A nonlinear relationship was observed between the two, with a turning point at 7.05 mg/kg/min. Improving IR might help mitigate the risk of AAC.

The eGDR is a marker of insulin sensitivity, strongly linked to metabolic syndrome, diabetes, and related complications (29). Lower eGDR values suggest IR (17). Our study highlights the association between eGDR and AAC, offering new insights into the connection between IR and atherosclerosis (30). Previous studies have also highlighted the nonlinear association between eGDR and the risk of cardiovascular disease mortality (14). Song et al. also further reported a significant negative relationship between eGDR and arterial stiffness, whereas the TyG index and HOMA-IR did not exhibit any meaningful correlation with arterial stiffness (21). According to the results of our ROC analysis, eGDR was more effective in predicting the risk of SAAC compared with other IR indices. The nonlinear relationship may reflect the complexity of the impact of IR on vascular calcification. Within a defined range, an increase in eGDR, which indicates improved insulin sensitivity, does not substantially decrease the risk of SAAC. However, a critical threshold appears to exist, beyond which increases in eGDR are associated with a marked reduction in SAAC risk. This threshold effect highlights the potential protective role of improved insulin sensitivity against vascular calcification, particularly in individuals transitioning from moderate to high eGDR levels. On the other hand, the more pronounced association in women may reflect sex-specific variations in hormonal and metabolic responses. Estrogen, particularly in premenopausal women, is recognized for its protective role in vascular health, potentially enhancing the beneficial effects of improved insulin sensitivity on vascular calcification (31, 32). This could explain the greater reduction in SAAC risk observed in women. Moreover, women may have heightened sensitivity to changes in insulin resistance-related pathways, which could magnify the influence of eGDR on AAC risk (33, 34). On the other hand, men may display a weaker association due to differences in hormonal regulation and vascular remodeling dynamics (31). In summary, our study establishes eGDR as the first IR marker to show both threshold-dependent and sex-specific associations with AAC risk. Unlike traditional IR metrics focused only on glycemic parameters, eGDR integrates central obesity and hypertension, offering a multidimensional measure of metabolic dysfunction and improving its utility in vascular calcification risk stratification. The identification of a specific eGDR threshold provides a clear target for intervention, while the stronger association in women challenges unisex approaches to cardiovascular prevention. These findings advance our understanding of IR-related vascular pathology, linking metabolic dysfunction to subclinical arterial disease, and position eGDR as a valuable tool for both epidemiology and clinical decision-making in precision medicine.

The relationship between IR, assessed via eGDR, and AAC, potentially mediated by various mechanisms. Both IR and AAC are strongly tied to chronic low-grade inflammation (35). In the context of IR, adipose tissue produces increased levels of pro-inflammatory mediators like TNF-α and IL-6, which can worsen endothelial dysfunction and accelerate vascular calcification (36, 37). IR can induce oxidative stress through lipotoxicity and glucotoxicity, and oxidative stress further promotes the expression of genes related to vascular calcification (35, 38). On the other hand, hyperglycemia, hyperinsulinemia, and lipid metabolism disturbances, which often accompany IR, may promote vascular calcification through pathways such as the formation of advanced glycation end products (AGEs) and lipid accumulation (39, 40). Insulin’s protective anti-inflammatory and anti-apoptotic effects are also reduced in IR, indirectly facilitating vascular calcification (41).

This study has some certain limitations. The cross-sectional design prevents the establishment of a causal relationship between eGDR and AAC. Furthermore, the reliance on the U.S. NHANES database and the inclusion of only adult participants reduce the external validity of the findings. Additionally, the omission of potential confounders, such as metabolic syndrome, metabolic-associated fatty liver disease (MAFLD), and the lack of diabetes classification might have introduced bias. Future research should aim to overcome these limitations by refining confounder control and conducting multicenter prospective studies to better understand this relationship.

## 5 Conclusion

In a study representative of the national adult population aged 40 and older, the eGDR was found to be linked to AAC risk. The eGDR holds potential as an epidemiological tool for assessing the impact of IR on AAC.

## Data Availability

Publicly available datasets were analyzed in this study. This data can be found here: https://www.cdc.gov/nchs/nhanes/.
